# Variations in microbial community compositions and processes imposed under contrast geochemical contexts in Sicilian mud volcanoes, Italy

**DOI:** 10.3389/fmicb.2024.1461252

**Published:** 2024-09-20

**Authors:** Jhen-Nien Chen, Yi-Ping Chiu, Tzu-Hsuan Tu, Francesco Italiano, Pei-Ling Wang, Li-Hung Lin

**Affiliations:** ^1^Department of Geosciences, National Taiwan University, Taipei, Taiwan; ^2^Department of Oceanography, National Sun Yet-sen University, Kaohsiung, Taiwan; ^3^National Institute of Oceanography and Applied Geophysics - OGS, Trieste, Italy; ^4^Institute of Oceanography, National Taiwan University, Taipei, Taiwan; ^5^Research Center for Future Earth, National Taiwan University, Taipei, Taiwan; ^6^Science and Technology Research Institute for Decarbonization, National Taiwan University, Taipei, Taiwan

**Keywords:** microbial community, methane, AOM, halophile, mud volcano, Sicily

## Abstract

Terrestrial mud volcanoes represent surface features of channels for subsurface methane transport and, therefore, constitute an important source of methane emission from natural environments. How microbial processes regulate methane emissions in terrestrial mud volcanoes has yet to be fully addressed. This study demonstrated the geochemical characteristics and microbial communities of four mud volcano and seep sites in two geological settings of Sicily, Italy. At sites within the accretionary wedge that exhibited higher methane and sulfate concentrations, the communities were dominated by members capable of catalyzing methane and sulfate metabolisms and organic degradation. In particular, both anaerobic and aerobic methanotrophs were abundant and their abundance distribution coincided with the geochemical transition. In contrast, the sites near Mount Etna were characterized by high fluid salinity, CO_2_, and low methane and sulfate concentrations, with communities consisting of halophilic organic degraders and sulfur metabolizers, along with a minor presence of aerobic methanotrophs. Substantial variations in community composition and geochemistry across spatial and vertical redox gradients suggest that physicochemical contexts imposed by the geology, fluid path, and source characteristics play a vital role in shaping community composition and cycling of methane, sulfur and organic carbon in Sicily mud volcanoes.

## 1 Introduction

Methane and CO_2_ are potent greenhouse gases. In addition to biological origins, geological features also contribute to the emission of both gases (Planke et al., 2003; Hensen et al., 2004; Mazzini, 2009; López-Rodríguez et al., 2019). Of all possible categories, mud volcanoes and seeps have been identified and estimated to release 10 of Tg yr.^−1^ of methane into the atmosphere (Etiope and Milkov, 2004; Etiope et al., 2007b), constituting one of the major sources of methane emissions from natural environments. While the transport of the geologically produced gases/fluids along the fracture system is primarily driven by the pressurization induced by depositional and tectonic loading, gas recharge, and density-controlled buoyancy at great depths (Dimitrov, 2002), both gases are susceptible to the addition and depletion through microbial processes near the surface (Niemann et al., 2006; Lösekann et al., 2007; Wang et al., 2014), thereby providing additional regulatory pathways for determining the quantity of ultimate emissions on a contemporary timescale.

Depending on the specific geological setting and generation mechanism, the relative abundance and isotopic composition of methane emitted from mud volcanoes and seeps vary widely. For the majority of previously investigated sites (>70%), the thermal maturation of organic matter at great depths accounts for the production of ^13^C-enriched methane and other higher hydrocarbon gases (C2+ gases, such as ethane, propane, and butane) (Etiope et al., 2009). In contrast, microbial processes are confined to shallower depths and represent the dominant methane generation process at only 4% of the previously investigated sites (Etiope et al., 2009). Because ^12^C-bearing precursors are preferentially utilized in the methane production, and microbially catalyzed production of higher hydrocarbons is circumstantial, microbial gases would be characterized by methane depleted in ^13^C and high methane-C2+ ratios (Conrad, 2009). Although thermal maturation and microbial processes generate distinct isotopic compositions of methane and methane-C2+ ratios, both yield gases with high CH_4_/CO_2_ ratios (>10). In contrast, gases from mud volcanoes or seep gases related to hydrothermal circulation or magmatic processes are enriched with CO_2_ (Wallace and Edmonds, 2011). While minor ^13^C-enriched methane or other higher hydrocarbons are recovered, the CH_4_/CO_2_ ratios of the emitted gases are often low (<0.01) (Etiope et al., 2007a). How microbial community assemblages and processes respond to distinct CH_4_/CO_2_ ratios and control the overall methane budget are not well understood.

Terrestrial mud volcanoes and seeps are distinct from their marine counterparts in many aspects. The main cause of these differences lies in a fact that bubbling pools and surrounding platforms in terrestrial mud volcanoes and seeps are directly exposed to the atmosphere. Once the fluid reaches the sediment-air interface, exsolved methane and carbon dioxide are discharged directly into the air (Etiope et al., 2008). Although microbial processes can profoundly alter methane fluxes and abundances in other ecosystems, only a limited number of studies have provided evidence to constrain the capacity and activity of methane consumption and production in terrestrial mud volcanoes and seeps. For example, it has been demonstrated that the removal of methane from depth is driven by the regeneration of electron acceptors (such as sulfate or iron oxyhydroxide) through the oxidation of reduced compounds associated with fluids that continuously or episodically emanate from mud pools in Taiwan (Chang et al., 2012; Cheng et al., 2012). The estimates of methane oxidation capacity in terrestrial mud volcanoes are, however, complicated by the presence of methanogenesis near sediment surface, through which the produced methane could be readily devoid of consumption catalyzed by anaerobic or aerobic methanotrophy (Wrede et al., 2012; Wang et al., 2014; Tu et al., 2017; Lin et al., 2018). Whether such interactions and distributive organization of methane production and consumption processes are universally valid for all terrestrial mud volcanoes regardless of CH_4_/CO_2_ ratios, remains largely unknown.

The Sicily Island of Italy is located at the boundary between the Eurasian and African Plates (Doglioni et al., 1999). Plate convergence has led to the gradual closure of the Tethys Sea and the formation of a series of fold-and-thrust belts distributed perpendicular to stress trajectories (Catalano et al., 2013). One of the major structural domains, the Apennine fold-and-thrust belt, extending from northern Italy to Sicily, is primarily composed of Neogene-Quaternary clastic sediments and represents the thin-skinned accretionary wedge (Monaco and De Guidi, 2006). Mud volcanoes and seeps related to such subduction related faulting are confined to the southwestern part of Sicily, with gases primarily composed of a mixture of microbial and thermogenic methane (>90%) with minor amounts of CO_2_ (several percentages), dinitrogen, higher hydrocarbons and cyclic compounds (Grassa et al., 2004; Tassi et al., 2012). In contrast, some mud volcanoes and seeps distributed at the foot of Mt. Etna are characterized by helium isotopic compositions indicative of mantle origin (Rizzo et al., 2006). Carbon dioxide represents the main gas phase (>90%) with isotopic compositions consistent with magmatic origin (Grassa et al., 2004). Minor amounts of thermogenic methane (<10%) and aromatic compounds have been detected (Tassi et al., 2012). The contrasting geological contexts and geochemical characteristics of mud volcanoes and seeps in Sicily provide an ideal opportunity to investigate how microbial communities respond to and whether specific methanotrophs and/or methanogens proliferate under such drastically different CH_4_/CO_2_ ratios.

This study aims to determine the microbial processes and community compositions in response to different geochemical contexts in terrestrial mud volcanoes and seeps in Sicily, Italy. Four sites located in eastern and southwestern Sicily were selected for investigation. To fully recover the microbial community assemblages, two sets of primers for archaeal and bacterial communities were tested individually. Finally, the molecular results were integrated with geochemical results reported previously (Tu et al., 2022) to address how geochemical contexts influence community composition and structure, and metabolism related to methane, sulfur, and carbon cycling.

## 2 Materials and methods

### 2.1 Site background and sample acquisition

Sicily is located at the boundary between the Eurasian and African Plates. The main structural feature related to the plate convergence is the Apennine-Maghrebian fold-and-thrust belt, which represents the accretionary wedge tectonically sandwiched between two imbricated blocks: the Panormide and Pelagian blocks (Catalano et al., 2013). The east–west trending Mount Kumeta-Alcantara fault system transects across the island and is terminated by the Siculo-Calabrian rift zone to the east. The petroleum systems of Sicily are related to the Apennine-Maghrebian fold-and-thrust belt and Pelagian foreland, where oil and thermogenic gases originating from the late Triassic-Early Jurassic source rocks were trapped (Tassi et al., 2012). Mud volcanoes and seeps are distributed in the eastern and southwestern parts of Sicily, with clastic sequences filling the system of Miocene to Pleistocene foredeep and piggy-back basins.

**Figure 1:**
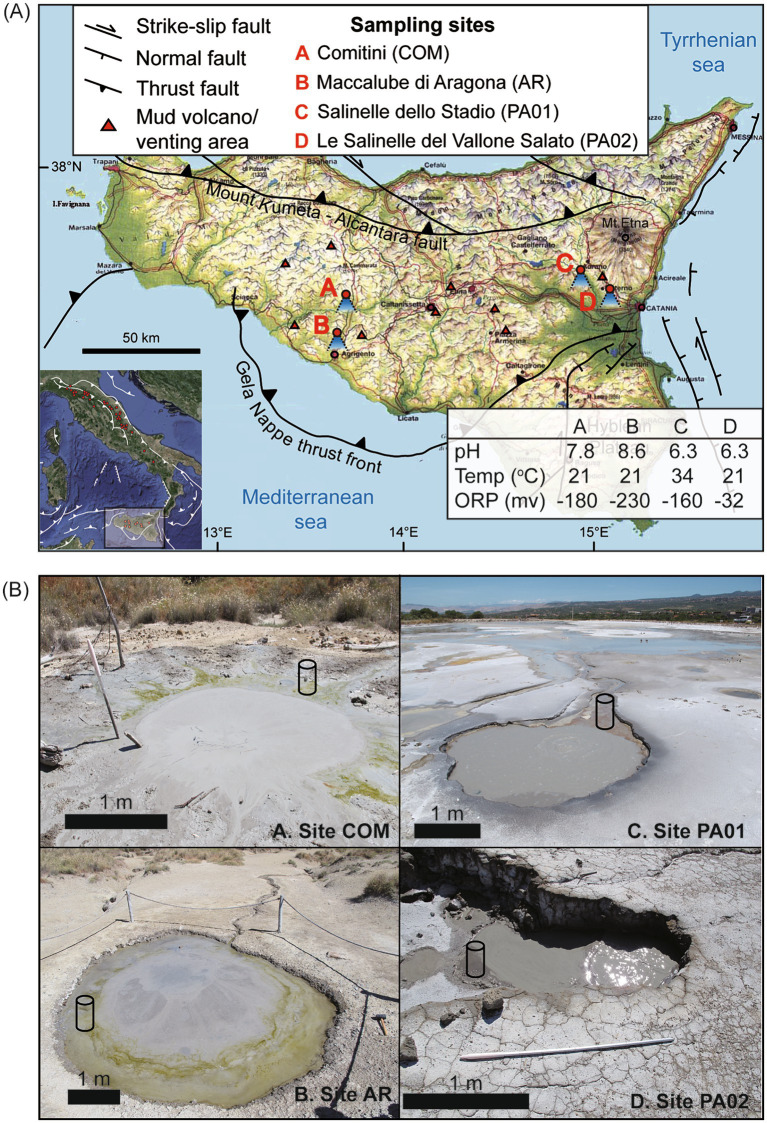
Topographic map **(A)** and field photos **(B)** for sampling sites of Sicily, Italy. Geological structures and general characteristics of sampled mud pools are provided in **(A)**. The exact locations of cores retrieved for analyses are marked with a cylinder symbol in **(B)**.

Samples were collected from four sites in the eastern and southwestern Sicily: Le Salinelle del Vallone Salato (PA01), Salinelle dello Stadio (PA02), Maccalube di Aragona (AR), and Comitini (COM) (Figure 1). The former two sites are distributed in the foothills south of Mt. Etna. Previous studies indicated that CH_4_/CO_2_ ratios from the region are mostly less than 0.2 (Grassa et al., 2004; Rizzo et al., 2006). The isotopic compositions and abundance ratios of alkanes indicate that these hydrocarbons were formed via thermal maturation of organic matter, potentially driven by magmatic intrusion at great depths (Grassa et al., 2004). Helium and CO_2_ possess isotopic compositions indicating a degassing from a magmatic source (Caracausi et al., 2003; Grassa et al., 2004; Rizzo et al., 2006). The integration of data suggests that gases formed at different depths merge at shallower depths and are transported via the deeply-rooted fault system toward the surface. In contrast, the latter two sites are distributed within the accretionary wedge between the Mount Kumeta-Alcantara fault system and Gella Nappe thrust front (Tassi et al., 2012). Gases are predominantly composed of methane, with minor hydrocarbons and CO_2_. The CH_4_/CO_2_ ratios range above 20 over time (Etiope et al., 2009). The isotopic compositions and abundance ratios of alkanes suggest that methane represents a mixture of microbial and thermogenic sources. The helium isotopic compositions indicate a crustal origin.

At individual sites, multiple mud pools with diameters ranging from 10 of centimeters to approximately 5 m were observed. Most mud pools featured continuous gas bubbling during the sampling period. At sites PA01 and PA02, salt crusts and orange microbial mats were widespread across the mud platform, suggesting that deeply sourced fluids were saline and reduced. Once the fluids are exposed to the atmosphere, evaporation and oxidation lead to the accumulation of halite and iron oxyhydroxides. At sites AR and COM, greenish layers (potentially algae) developed along the fringes of several mud pools (Figure 1B).

Two types of samples, mud pool fluids and cored sediments, were collected. Fluids with muds at approximately 10 cm below the air-water interface at the center of the mud pool were scooped with a sterilized polypropylene (PP) cup. For the cored sediments, a sterilized PP liner was inserted at the fringe of the mud pool (Figure 1B). Once retrieved, the core liner was capped on both sides immediately to prevent oxygen intrusion from the core bottom. All core samples were well preserved during the transportation and further processed in the lab within 6 h of collection.

Before the sample collection, environmental parameters (including pH, temperature, and redox potential) of the pool fluids were measured using probes connected to a handheld multi-purpose meter (WTW, Germany). All the sensors were calibrated before the field trip.

### 2.2 Sample processing and analyses

Cored sediments were sectioned at a 2–3 cm interval using sterilized knives. The sectioned sediments and pool fluids were further processed for various analytical purposes. For gas samples, 6 mL of sediment/pool fluid was retrieved using syringes and injected into serum bottles filled with 10 mL of 1 M NaOH. Serum bottles were sealed with thick butyl rubber stoppers. About 40 mL of sediments/fluids was placed into sterilized centrifuge tubes and spun at a speed of ~6,000 g for 20 min. The spun sediments and intact pool fluids were preserved at −20°C for short-term storage and − 80°C for long-term storage for molecular analyses. The supernatant of the spun pool fluids and sediments was filtered using a 0.22 μm pore-sized membrane. The filtrate was further split into several fractions for analysis. For all samples, 2 mL of pore fluid was stored for cation (treated with 1% nitric acid) analysis. Because not all samples contained a sufficient volume of pore fluids, analyses of dissolved inorganic carbon (DIC) were conducted on samples from all depths for the AR core, the top 27 cm for the COM core, and the top 32 cm for the PA02 core. For this purpose, 2 mL of pore fluid was injected into a 10 mL pre-sealed serum bottle filled with 0.5 mL 85% phosphoric acid and nitrogen gas. Portions of the sectioned sediments and pool fluids were weighed and freeze-dried. The loss of mass was used to calculate the water content or porosity, assuming that the density of the dry sediment was 2.5 g cm^−3^ and the pore space was completely saturated with pore fluid. The dried sediments were ground and homogenized for elemental analysis.

The abundances of hydrocarbons and DIC were analyzed using a gas chromatography equipped with a Porapack Q column, a thermal conductivity detector, and a flame ionization detector (6,890 N, Agilent Taiwan, Taiwan). The measured partial pressure was converted into the dissolved concentration by summing the total moles of the target molecules in the headspace and solution and dividing this value by the volume of pore fluid preserved in the sampled vials. The carbon isotopic compositions were analyzed using a gas chromatography (GC IsoLink, Thermal Fisher Scientific) in line with a Finnigan MAT253 isotope ratio mass spectrometry. The isotopic compositions were reported using *δ* notation [δ value = (R_sample_/R_std_ − 1) × 1,000 ‰, where R is the ^13^C/^12^C ratio and std. is the Vienna Pee Dee Belemnite (VPDB)]. Soluble iron and manganese were measured using an inductively coupled plasma-optic emission spectrometer (ICP-OES) with a detection limit of 2 ppm (Perkin Elmer, United States). The uncertainties for aqueous chemistry were ±2%, whereas that for δ^13^C-DIC was ±0.3%. In addition to the analyses mentioned above, we incorporated the geochemical data reported by Tu et al. (2022) for further interpretation and discussion. These data include the concentrations of chloride, sulfate, methane and δ^13^C values of methane, as well as the concentrations of particulate total organic carbon (TOC), total inorganic carbon (TIC), total nitrogen (TN) and total sulfur (TS).

Two representative cores collected from sites AR and PA02 were used for gene analyses. This selection was based on the geochemical data reported by Tu et al. (2022) and this study, in which the sites AR and COM could be categorized from the other cluster encompassing sites PA01 and PA02. Furthermore, the community analysis in Tu et al. (2022) also shows that the communities for sites AR and COM resemble to each other at a higher degree than to those for sites PA01 and PA02 (see more details in complementary text). These selected samples included pool fluid and sediments at 1.5, 11.5, 14.5, 17.5, 29.5, 41.5, 53.5 and 65.5 cm depth from site AR; pool fluids and sediments at 0.8, 5, 12.8, 22.8, 31, and 39 cm depth from site PA02. Previous studies have attested that community compositions in heterogenous environments (e.g., soil system) can vary considerably at small sample size (Ranjard et al., 2003), leading to the biased interpretation of community pattern. Resolving such highly heterogeneous community compositions cannot be matched parallelly by the porewater or sediment geochemistry primarily because of the limit in methodological resolution and sensitivity. The resolution of both molecular and geochemical data sets could be better comparable if the sample size for molecular analyses is enhanced (Ellingsøe and Johnsen, 2002; Penton et al., 2016). Therefore, to minimize the potential bias caused by the sample heterogeneity, we increased the sample size to 10 g sediments for DNA extraction to average out the potential heterogeneity of community compositions attributed to the conventional size (<1 g) adopted by most commercial kits. Crude DNA was extracted using the MoBio Ultraclean Mega DNA Prep Soil Kit (MoBio, USA) according to the manufacturer’s instructions and stored in water at −80°C.

Previous studies have demonstrated that different combinations of primers could lead to substantial distortion on community composition (Claesson et al., 2010; Tremblay et al., 2015), as well as richness, and diversity evaluation (He et al., 2013), even though primer specificity has been examined *in silico* (Acinas et al., 2005). To avoid this during polymerase chain reaction (PCR), two sets of primers were used to amplify the bacterial and archaeal communities individually (Table 1). These primer sets targeted V1-V3 (VB01 set) and V3-V6 (VB02 set) regions for bacterial communities, and V3-V5 (VA01 set) and V4-V6 (VA02 set) regions for archaeal communities. The PCR schemes included a denaturation step at 94°C for 1 min, followed by 20 (for bacteria) or 23 (for archaea) cycles of denaturation at 94°C for 30 s, annealing at 58°C (for bacteria) or 54°C (for archaea) for 30 s and extension at 72°C for 1 min, and a final extension step at 72°C for 7 min. Five replicates of PCR reaction were conducted on individual samples. The PCR products were checked using gel electrophoresis, pooled, and purified. A second PCR (5 cycles) with primers consisting of the original primer in the first PCR, barcode (6 mer), key, and adapter was applied to the purified PCR product. The product of the second PCR was purified and quantified for its concentration using a Qubit dsDNA HS Assay kit (Invitrogen, USA). Equal amounts of PCR products from different samples were pooled and sequenced on a Roche 454 GS Junior platform (Roche/454 Life Sciences, USA).

**Table 1:** Primers and their target genes/taxonomic groups for analyses of community compositions and gene abundances.

Primer set	Primer name	Sequence (5′-3′)	Purpose	Target group/gene	Annealing temp (°C)	Reference
VB01	B27F	AGAGTTTGATCMTGGCTCAG	NGS	Bacteria/16S rRNA	58	Lane (1991)
	E517R	TTACCGCGGCTGCTGGC	NGS	Bacteria/16S rRNA	58	Sikorski et al. (2001)
VB02	E517F	GCCAGCAGCCGCGGTAA	NGS	Bacteria/16S rRNA	58	Liu et al. (2007)
	E1046R	CGACAGCCATGCANCACCT	NGS	Bacteria/16S rRNA	58	Sogin et al. (2006)
VA01	A340F	CCCTACGGGGYGCASCAG	NGS	Archaea/16S rRNA	54	Takai and Horikoshi (2000)
	A912R	CCCCCGCCAATTCCTTTAA	NGS	Archaea/16S rRNA	54	Miyashita et al. (2009)
VA02	A519F_mpi	CAGCMGCCGCGGTAA	NGS	Archaea/16S rRNA	54	Teske and Sørensen (2008)
	A1041R_mpi	GGCCATGCACCWCCTCTC	NGS	Archaea/16S rRNA	54	Baker et al. (2003)
	A8F	TCYGGTTGATCCTGCC	qPCR	Archaea/16S rRNA	54	Huber et al. (2002)
	U1513R	ACGGHTACCTTGTTACGACTT	qPCR	Archaea/16S rRNA	54	Huber et al. (2002)
	U1492R	CGGTTACCTTGTTACGACTT	qPCR	Bacteria/16S rRNA	54	Lane (1991)
	Eub338R	GCTGCCTCCCGTAGGAGT	qPCR	Bacteria/16S rRNA	48	Amann et al. (1990)
	Arch806F	ATTAGATACCCSBGTAGTCC	qPCR	Archaea/16S rRNA	53	Takai and Horikoshi (2000)
	A958R	YCCGGCGTTGAMTCCAATT	qPCR	Archaea/16S rRNA	53	Hinrichs et al. (1999)
	ANME1-350F	GCATCAGGCGCGAAAACT	qPCR	ANME-1/16S rRNA	63	Boetius et al. (2000)
	ANME1-1417R	CCTCACCTAAACCCCACT CCTCACCTAAATCCCACT	qPCR	ANME-1/16S rRNA	63	Miyashita et al. (2009)
	ANME2-712F	GGGACCATCTGTGGCGAA	qPCR	ANME-2/16S rRNA	57.5	Knittel and Boetius (2009)
	EelMS932R	AGCTCCACCCGTTGTAGT	qPCR	ANME-2/16S rRNA	57.5	Boetius et al. (2000)
	amoA-1F	GGGGTTTCTACTGGTGGT	qPCR	AOB/*amoA*	54	Rotthauwe et al. (1997)
	amoA-2R	CCCCTCKGSAAAGCCTTCTTC	qPCR	AOB/*amoA*	54	Rotthauwe et al. (1997)
	Camoa-19F	ATGGTCTGGYTWAGACG	qPCR	AOA/*amoA*	55	Pester et al. (2012)
	CamoA-616R	GCCATCCABCKRTANGTCCA	qPCR	AOA/*amoA*	55	Pester et al. (2012)

Sequence reads were first processed by correcting sequence errors and filtering low-quality and short reads using the software Mothur 1.29 (Schloss et al., 2009). The trimmed reads were further denoised and checked for chimera formation. The unique reads were aligned to the SILVA SSU dataset of the NR 128 release^1^ and classified into different operational taxonomy units (OTUs) based on 97% identity using the average neighbor algorithm (Schloss and Westcott, 2011). The taxonomy was assigned based on a naïve Bayesian classifier using the k-nearest neighbor consensus method (Wang et al., 2007). The entire process followed a standard protocol (Schloss and Westcott, 2011). To avoid the diversity inflation associated with sequence numbers, diversity indices were calculated from the dataset that was subject to the random resampling of reads down to a level for the sample with the lowest read number. To compare community structures among samples, principal coordinate analyses (PCoA) were conducted based on Bray–Curtis dissimilarity (Bray and Curtis, 1957). The sequence data of the unique OTUs were submitted to NCBI (BioProject ID: PRJNA735407).

Genes subjected to quantitative PCR (qPCR) analyses included 16S rRNA genes of bacterial, archaeal, and ANME-1 and -2 groups, as well as the gene encoding ammonia monooxygenase subunit A (amoA). The *amo*A gene was selected to detect ammonium oxidizers, because ammonium may accumulate when sediment organic matters are degraded (Lin et al., 2014). Analyses were performed on a MyiQ Real-Time PCR Detection System (Bio-Rad, USA). Each qPCR reaction contained 1X SsoFast EvaGreen Supermix (Bio-Rad, USA), 100 nM of each primer, and 2 μL of the template. The primers and target groups included 806f/958r for archaea, B27f/EUB338r for bacteria, ANME1-350F/ANME1-1417R for ANME-1, ANME2-712F/EelMS-932R for ANME-2, amoA-19F/amoA-2R for bacterial amoA (AOB), and Camoa-19F/CamoA-616R for archaeal amoA (AOA) (Table 1). Each qPCR temperature program started with a denaturation step at 98°C for 5 min, followed by 40 cycles of 30 s denaturation at 98°C and 40 s for annealing. The annealing temperature for each primer pair is listed in Table 1. Primer specificity was confirmed by melting curve analysis and gel electrophoresis. Standards for bacterial and archaeal 16S rRNA genes were prepared from nearly full-length sequences amplified from the crude extracts using domain-specific primers (B27F/U1492R for bacteria and A8F/U1513R for archaea) (Lane, 1991; Huber et al., 2002). The standards for ANME-1 and -2 16S rRNA genes and bacterial and archaeal *amo*A genes were prepared from cloned amplicons of these target groups. The concentration of standard DNA was determined using a Qubit spectrophotometer (Invitrogen, USA). The gene copy number was calculated assuming 650 g/mol of one base pair of DNA.

## 3 Results

### 3.1 Fluid characteristics

**Figure 2:**
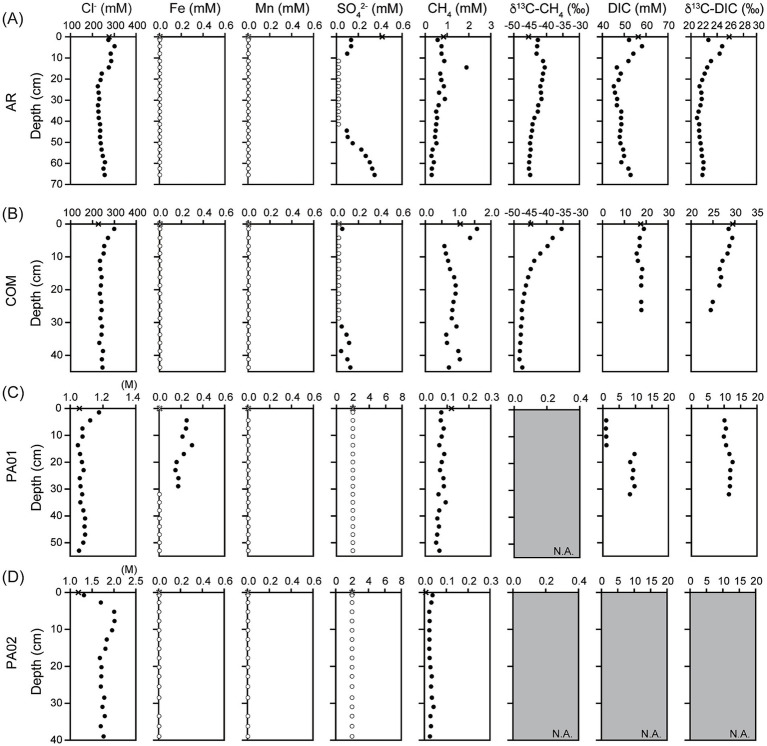
Geochemical characteristics of fluids collected from mud pools and sediments from **(A)** Aragona (AR), **(B)** Comitini (COM), **(C)** Paterno (PA01), and **(D)** Vallone (PA02). Cross symbols marked at 0 cm depth represent the concentrations or isotopic compositions of fluids from mud pools. Open circles represent the concentrations below the detection limit. Panels filled with gray color and marked with N.A. indicate that the data is not available because the volume or concentration was too low for analyses. Data for the concentration of chloride, sulfate, methane and δ^13^C-methane were adopted from Tu et al. (2022).

The geochemistry data obtained in this study were combined with those reported in Tu et al. (2022) to comprehensively address the variation patterns along depth or across sites in Sicily. All investigated mud pools were continuously bubbling with patchy black oily materials on the pool surface during sampling (Figure 1). General characteristics of fluids were, however, dependent on geographic regions. At site AR, the pool fluids were alkaline (pH 8.6) and reducing (Eh −230 mV). DIC concentrations were 56.4 mM for pool fluids and ranged between 45.3 mM and 58.2 mM for pore fluids. The δ^13^C values of DIC were 25.8‰ for pool fluids, and ranged between 20.9‰ and 24.8‰ for pore fluids. Ethane and propane concentrations ranged between 0.42 and 4.73 μM and between 0.05 and 1.35 μM, respectively (Supplementary Figure S1), and varied in a fashion similar with DIC concentrations. Dissolved Fe and Mn concentrations were below the detection limit (Figure 2A).

The geochemical characteristics of samples from site COM were generally comparable to those from site AR. The pool fluids were slightly alkaline (pH 7.8) and reducing (Eh −180 mV) (Figure 2B). Due to the limited volume available for analysis, DIC concentrations were obtained only for the top 26.3 cm. Its concentrations ranged between 16.2 and 18.9 mM and exhibited insignificant variation along depth. The δ^13^C values of DIC, however, decreased from 30‰ in the pool fluids and top sediments to 24.5‰ at 26.3 cm. Ethane and propane concentrations were mostly below 2 and 0.2 μM, respectively, and did not exhibit any systematic variation with depth (Supplementary Figure S1). Dissolved Fe and Mn concentrations were below the detection (Figure 2B).

The geochemical characteristics at sites PA01 and PA02 were in great contrast to those at sites AR and COM. Pool fluids at site PA01 were slightly acidic (pH 6.3) and oxidizing (Eh −32 mV) (Figure 2C). DIC concentrations were low (~1 mM) at shallow depths (<13.8 cm) and increased to between 8.3 and 9.7 mM at greater depths. The concentration transition corresponded to a shift in carbon isotopic composition from 10.3 to 11.8‰ (Figure 2C). Notably, the dissolved iron was detected at 0–30 cm but absent in the other samples (Figure 2). Ethane and propane concentration were at μM and sub-μM scales (Supplementary Figure S1). The geochemical characteristics at site PA02 were comparable to those at site PA01. Ethane concentrations decreased from 0.51 μM in top sediments to 0.087 μM at core bottom (Supplementary Figure S1). Propane was undetectable. Dissolved iron and manganese concentrations were below the detection limit (Figure 2D).

### 3.2 16S rRNA gene diversities and community assemblages

A total of 209,579 raw reads of 16S rRNA gene amplicons were obtained (Supplementary Table S1). After sequence denoise, chimera removal, and quality screening (Q_20_), 205,254 reads with average lengths between 463 and 482 bps were acquired for further clustering, diversity, taxonomic, and community analyses. Using 97% sequence similarity as a cutoff, a total of 1,309 and 1,428 archaeal OTUs and 2,072 and 1,670 bacterial OTUs were obtained, depending on the primer pairs used. The diversity and community composition exhibited drastically different patterns at different sites.

At site AR, the sequences (46,678 archaeal and 40,566 bacterial sequences; Supplementary Table S1) were further clustered into 514 and 747 archaeal OTUs for amplicons generated from the VA01 and VA02 primer pairs, and 1,043 and 836 bacterial OTUs for amplicons generated from the VB01 and VB02 primer pairs, respectively. The sequencing depth was translated into at least 91% coverage for all samples (Supplementary Figure S2). The Chao1 indices for archaeal communities ranged between 100 and 150 and exhibited a moderate dependence on depth regardless of the primers used (Supplementary Figure S2A). For comparison, the Chao1 indices for bacterial communities decreased from 800 to 570 at <15 cm, remained at ~620 at depth for amplicons generated by the VB01 primer set, and varied within a small range of 500 ± 30 for amplicons generated by the VB02 primer set (Supplementary Figure S2B). Most Shannon indices varied in a fashion comparable with the Chao1 indices with the exception that the VA01 primer set generated fluctuated indices along depth (Supplementary Figure S2).

**Figure 3:**
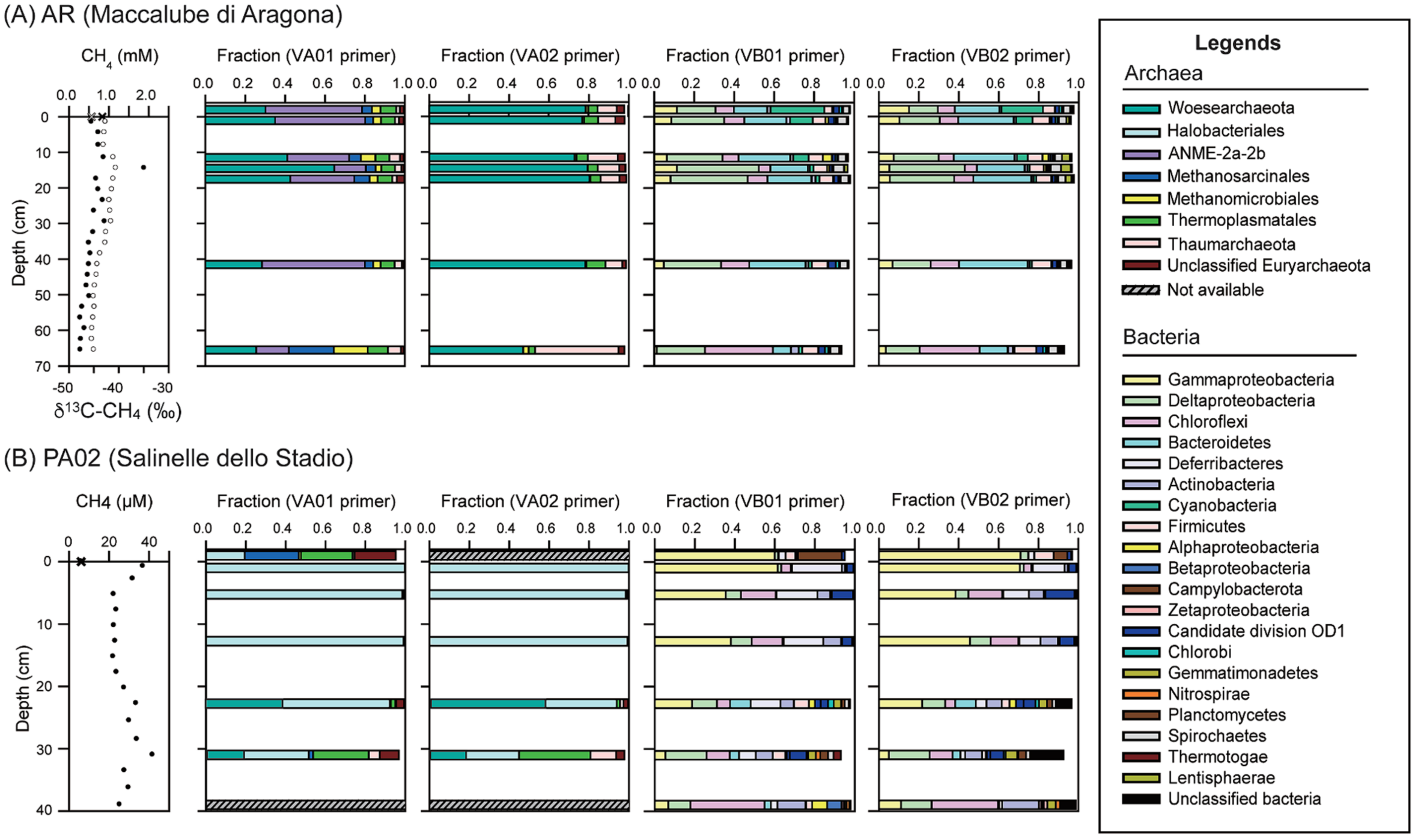
Community compositions based on 16S rRNA gene amplicons for samples from Aragona **(A)** and Paterno **(B)**. Methane concentrations (solid circles) and isotopic compositions (open circles; only for site Aragona) in the left panel are provided to constrain the possible methane metabolisms. Two primer sets (see Table 1 for detailed information) were used to amplify individual bacterial and archaeal communities of each sample. Archaeal community compositions for the mud pool and bottom sediments at Paterno could not be obtained because the DNA concentrations were too low.

A total of 16,352 archaeal reads were obtained using the VA01 primer set (Supplementary Table S1). Of various taxonomic groups, phylum Woesearchaeota (33.1%), order Thermoplasmatales (7.1%), Methanosarcinales (excluding ANME-2a-2b) (6.5%) and Methanomicrobiales (5.5%), and family ANME-2a-2b (36.9%), predominated over the other archaeal members (Figure 3; Supplementary Table S2). While their summed abundances exceeded 89% of the total reads, their individual abundances varied with depth. The abundances of Woesearchaeota related sequences increased from 25.2% in pool fluids to 29.3% at 1.5 cm, peaked at 14.5 cm (60.1%), and declined with depth (23.3% at core bottom). The majority of these Woesearchaeota (93%) were composed of uncultured Pacearchaeota members (former DHVEG-6) (Castelle et al., 2015). The ANME-2a-2b groups constituted the second largest group. Their abundances ranged between 15.9 and 51.0% with the lowest value at 14.5 cm and elevated values at shallower and greater depths. The variation of ANME-2a-2b along depth was negatively correlated with that of Woesearchaeota (R^2^ = −0.5). The abundances of Methanosarcinales (excluding ANME-2a-2b) and Methanomicrobiales increased with depth in a similar fashion. Their individual abundances reached up to 17–23% of the total archaeal sequences at the core bottom. *Methanosaeta* and *Methanocalculus* represented the largest genera and constituted more than 80% of the individual orders. Thermoplasmata and Thaumarchaeota were the minor community components (<7%) and varied at a small magnitude along depth.

A total of 30,326 archaeal sequences were obtained using the VA02 primer set (Supplementary Table S1). Woesearchaeota and Thaumarchaeota were the most abundant phyla, being represented by 70.8 and 8.0% of total reads, respectively (Figure 3). The abundances of Woesearchaeota remained at a nearly constant level (73–77%) above 41.5 cm and decreased to 44.2% at 65.6 cm. Like those obtained from the VA01 primer set, the Pacearchaeota members predominated over the others (98%; Figure 3). Thaumarchaeota varied in an opposite fashion from that of Woesearchaeota (R^2^ = −1) with the highest abundance (19.6%) at 65.5 cm. Sequences belonging to this phylum were classified to uncultured Lokiarchaeota (formerly MBG-B) (Spang et al., 2015), Miscellaneous Crenarchaeota Group (MCG), and Group C3. The abundances of Lokiarchaeota related members were great (7.3 and 6.0% of the total) at 11.5 and 14.5 cm, whereas those of MCG and C3 related members varied insignificantly at shallow depths and increased to more than 10% of the total at 65.5 cm. Sequences related to anaerobic methanotrophs and methanogens constituted only a minor fraction of the archaeal communities (<2%).

A total of 19,046 sequences were obtained using the VB01 primer set (Supplementary Table S1). The phyla and classes with an abundance cutoff of 1% included Deltaproteobacteria (31.3%), Bacteroidetes (21.5%), Chloroflexi (14.1%), Gammaproteobacteria (7.9%), Firmicutes (5.8%), Spirochaetes (2.1%), OD1 (Parcubacteria) (2.6%), Actinobacteria (2.0%), Alphaproteobacteria (1.3%) (Figure 3). The abundances of Deltaproteobacteria ranged from 25.9% in the mud pool to 29.8% at 11.5 cm, peaked at 41.4% at 14.5 cm, and decreased with depth. Within Deltaproteobacteria, the fermentative Bdellovibrionales (5.7%), sulfate-reducing Desulfobacterales (28.9%), Desulfarculales (4.7%), and metal-reducing Desulfuromonadales (56.8%) predominated over the others. The abundances of Bacteroidetes were high (19–28%) at most depth intervals and decreased to 9.1% at 65.5 cm. The abundances of Chloroflexi ranged between 5 and 14% above 41.5 cm and increased to 34.2% at 65.5 cm, while those of Firmicutes ranged between 5 and 6% in sediments and 3.9% in the mud pool. The abundances of Gammaproteobacteria were 4–15% above 41.5 cm and decreased to 1.3% at 65.5 cm. Within Gammaproteobacteria, family Methylococcaceae with members composed of aerobic methanotrophs (Kumar et al., 2021) appeared to be abundant (2–3%) in the mud pool and top sediments, and sparse at deeper intervals.

A total of 21,520 sequences were obtained using the VB02 primer set. The abundances and variation patterns of the major phyla were highly comparable with those obtained using the VB01 primer set (Figure 3).

At site PA02, the sequences (33,991 archaeal and 29,933 bacterial sequences; Supplementary Table S1) were further clustered into 815 and 690 archaeal OTUs for amplicons generated from the VA01 and VA02 primer sets, and 1,066 and 879 bacterial OTUs for amplicons generated from the VB01 and VB02 primer sets, respectively. Such sequencing depth was translated into at least 82% coverage for all samples (Supplementary Figure S2). The Chao1 index for archaeal communities ranged between 46 and 283, whereas the evenness index ranged between 0.59 and 0.88. Both indices varied with depth and were higher at depth. For comparison, the Chao1 index for the bacterial communities ranged between 139 and 983, whereas the evenness index ranged from 0.36 to 0.82. Both indices exhibited an increase as the depth increased to 31 cm and decreased at the core bottom regardless of the primer set used. Most Shannon index varied in a fashion comparable to that of the Chao1 index (Supplementary Figure S2).

A total of 21,741 archaeal sequences were obtained using the VA01 primer set (Supplementary Table S1). Woesearchaeota (29.7%) and Euryarchaeota predominated over other archaeal members and were primarily composed of Halobacteriales (59.8%), Thermoplasmatales (7.5%), Methanosarcinales (7.6%; excluding ANME related groups), and unclassified archaea (2.7%) (Figure 3). Family ANME-2a-2b and class Methanomicrobiales constituted less than 0.5% of the archaeal communities. Of all the major detected groups, the abundance of Halobacteriales-related members was 10.3% in the mud pool and decreased from >96% in the top sediments to 27.6% at 31 cm depth. Within Halobacteriales, the family Halobacteriaceae (83%) predominated over Pacearchaeota members in sediments. In contrast, both groups shared comparable abundances (9–10%) in the mud pool. The abundances of Thermoplasmatales and Methanosarcinales varied inversely with those of Halobacteriales. Both these orders shared comparable abundances in the mud pool (20–27%). Within the order Methanosarcinales, genera *Methanosaeta* and *Methermicoccus* were more abundant. Their abundances were much greater in the mud pool (11–15%) than in the sediments (<2%).

A total of 25,205 archaeal sequences were obtained using the VA02 primer set. The compositions and variation patterns were comparable with those obtained using the VA01 primer set with the exception that ANME-2a-2b and Methanomicrobiales were not detected, and Methanosarcinales was extremely rare (one sequence).

A total of 21,554 sequences were obtained using the VB01 primer set. The major phyla detected included Gammaproteobacteria (35.3%), Deferribacteres (11.5%), Chloroflexi (11.1%), Deltaproteobacteria (8.4%), Actinobacteria (1.6%), Bacteroidetes (2.8%), Firmicutes (1.5%), OD1 (1.8%), Gemmatimonadetes (1.3%) (Figure 3). The abundances of Gammaproteobacteria decreased with increasing depth. Within Gammaproteobacteria, Methylococcales, Thiotrichales, and unclassified *Thiohalophilus* and *Thiohalorhabdus* represented the major taxonomic groups, constituting 88% of the total Gammaproteobacteria sequences. The abundances of Deferribacteres were low (3.4%) in the mud pool, reached 25% in the top sediments, and decreased with depth in the sediments. Chloroflexi abundances increased from 0.1% in the mud pool to 17.5% at 5.3 cm, decreased to 6.6% at 22.8 cm and increased again to 37.1% at the core bottom. The class Anaerolineae constituted 74% of the total Chloroflexi reads. The abundances of Actinobacteria increased from less than 1% in the mud pool to 14.1% at 39 cm. The abundances of Deltaproteobacteria increased from 1.6% in the mud pool to 20.5% at 31 cm, and declined to 11.0% at 39 cm. The sulfate-reducing orders Desulfobacterales and Desulfarculales appeared to constitute the majority of sequences related to this class.

A total of 27,591 bacterial sequences were obtained using the VB02 primer set. Again, the abundance and variation patterns of individual phyla and lower taxonomic units were highly comparable to those obtained using the VB01 primer set.

### 3.3 Gene abundances

**Figure 4:**
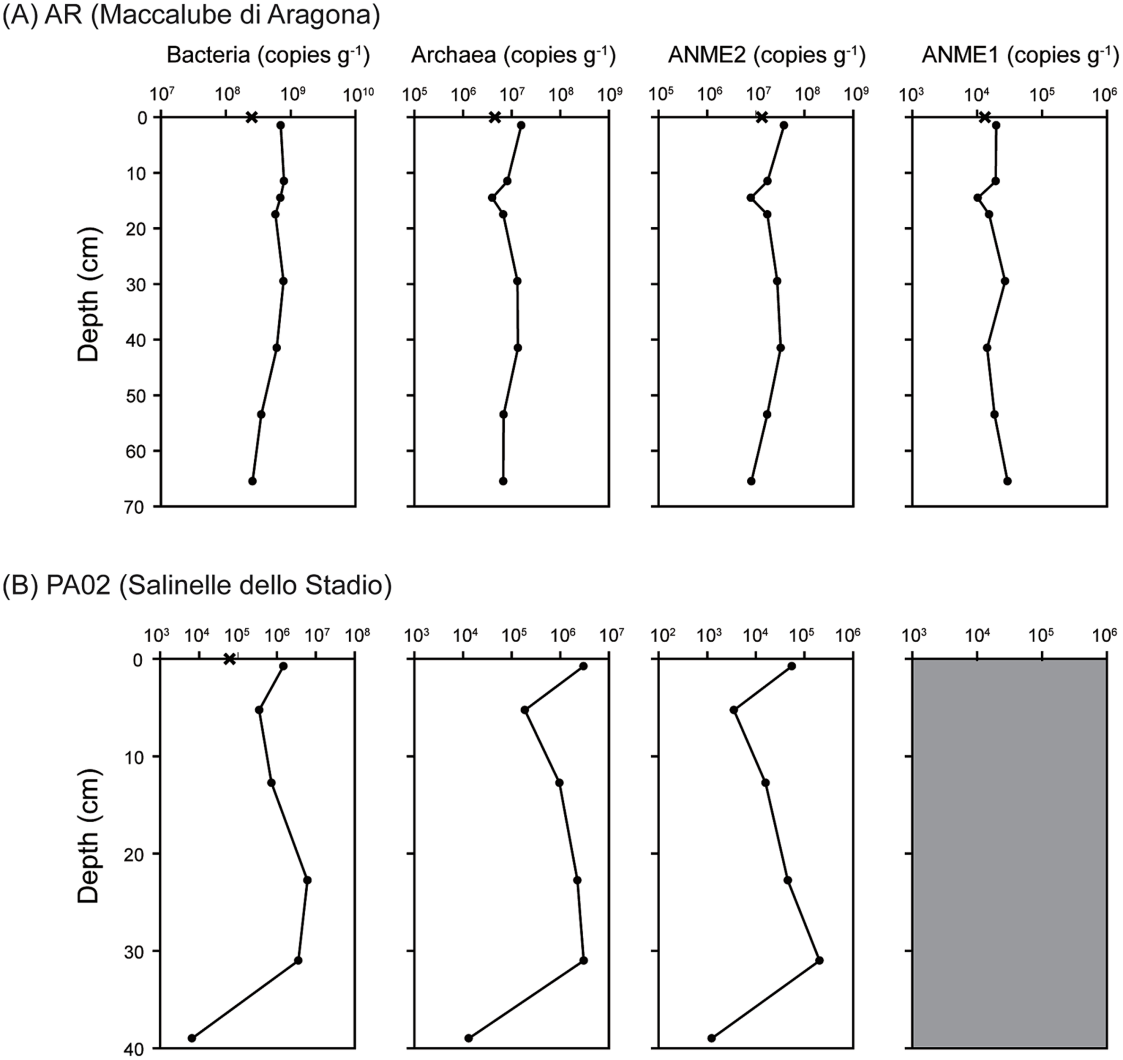
Copy numbers of 16S rRNA gene for different taxonomic groups. **(A)** Results for site Agragona (AR). **(B)** Results for site Vallone (PA02). All the reported values represent the averages of triplicate analyses. The standard deviations were less than 20% of the average values and too small to be shown. The gray area for the ANME1 group from site PA02 represents the gene abundances were below the detection level.

The qPCR analyses yielded different variation and abundance patterns for different taxonomic groups at different sites (Figure 4). AOA and AOB were both below the detection limit at the two sites. At site AR, the 16S rRNA gene abundances of bacteria were 2.50 × 10^8^ copies (g^−1^ fluid) in the mud pool and ranged between 2.57 × 10^8^ and 7.88 × 10^8^ copies (g^−1^ sediment) in sediments (Figure 4A). The abundances peaked at 11.5 cm and decreased generally with depth. The 16S rRNA gene abundances of archaea were 4.50 × 10^6^ copies (g^−1^ fluid) in the mud pool, and ranged from 3.97 × 10^6^ to 1.57 × 10^7^ copies (g^−1^ sediment) in sediments with the lowest value at 14.5 cm. The 16S rRNA gene abundances of ANME-1 ranged from 1.02 × 10^4^ to 2.96 × 10^4^ copies (g^−1^ sediment), whereas the 16S rRNA gene abundances of ANME-2 were at least two orders of magnitude greater than ANME-1 and ranged from 7.94 × 10^6^ to 3.80 × 10^7^ copies (g^−1^ sediment). The variation patterns of both ANME-1 and ANME-2 gene abundances were comparable to those of archaea.

For site PA02, the gene abundances of all investigated groups varied in a similar fashion with the exception that ANME-1 abundances were below the detection (Figure 4B). Bacterial 16S rRNA gene abundances were 6.10 × 10^4^ copies (g^−1^ sediment) in the mud pool, decreased from 1.46 × 10^6^ copies (g^−1^ sediment) in the top sediments to 3.48 × 10^5^ copies (g^−1^ sediment) at 5.3 cm, increased to 6.09 × 10^6^ copies (g^−1^ sediment) at 22.8 and 31.0 cm, and decreased again to 6.51 × 10^3^ copies (g^−1^ sediment) at 39 cm. Archaeal and ANME-2 16S rRNA gene abundances ranged between below the detection (in the mud pool) and 3.01 × 10^6^ copies (g^−1^ sediment). Both followed a variation trend similar to that of the bacteria.

### 3.4 PCoA analysis

**Figure 5:**
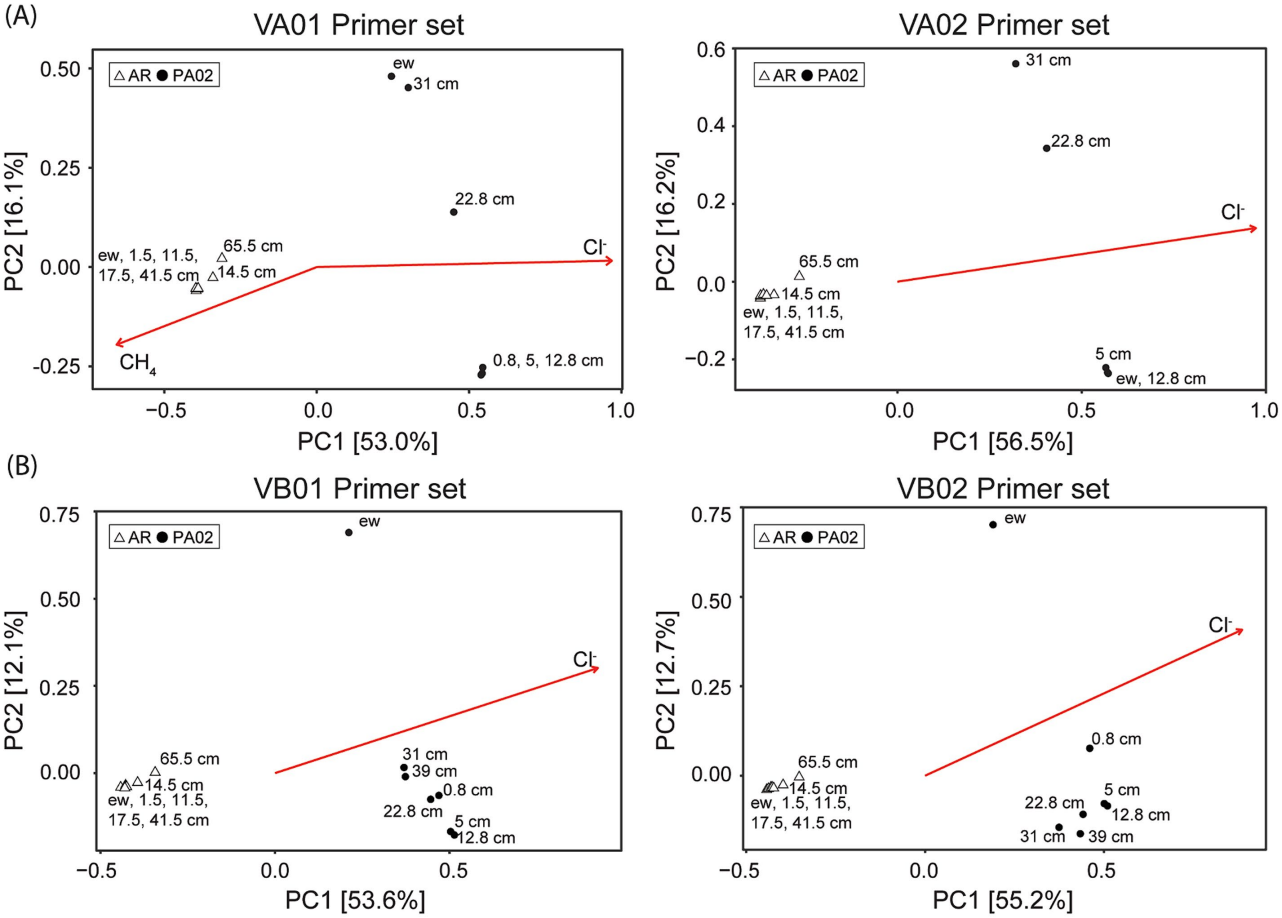
Community variance based on PCoA analysis using different primer sets. **(A)** Archaeal community variance. **(B)** Bacterial community variance. The open triangles represent the communities for site AR, whereas the solid circles represent the communities for site PA02. Ordination of significant geochemical parameters is overlaid for comparison. The detailed information for individual primer set is described in Table 1.

Principal coordinate analyses (PCoA) based on the Bray–Curtis dissimilarity matrix yielded a clear distinction of community patterns between sites and within sites regardless of the primer sets adopted (Figure 5). For archaeal communities, PC1 and PC2 explained 53–56 and 16% of community variance, respectively (Figure 5A). Communities recovered from site AR were clearly distinguished from those recovered from site PA02 mostly by PC1. However, communities from individual sites exhibited varying degrees of PC2 variation. The communities recovered from the bottom intervals (65.5 cm for site AR, and 22.8 and 31.0 cm for site PA02) deviated from the cluster composed of communities at shallower intervals. Similarly, bacterial communities from site AR were distinguished from those from site PA02 by PC1 (Figure 5B). Again, variance primarily along PC2 existed for communities within individual sites. Bacterial communities from site AR clustered tightly in the second quadrant, whereas those recovered from site PA02 varied considerably over a wide region of the first and fourth quadrants. In particular, the mud pool community deviated from the others, forming a loose cluster regardless of the primer set used. Of all selected environmental factors (chloride, sulfate and methane), chloride significantly contributed to the overall differences in community composition regardless of the primer sets, while methane was significantly correlated to the community variance with VA01 primer set (Figure 5).

## 4 Discussion

### 4.1 Origin of fluids/sediments and surface alteration

Mud pools represent the surface expression of fluid conduits that tap deep fluid/hydrocarbon reservoirs (Dimitrov, 2002; Etiope et al., 2009). As the fluids/sediments emanate off the mud pool and accumulate in the adjacent mud platform, geochemical and mineralogical characteristics and microbial communities and activities would be subject to modifications caused by surface processes (Chang et al., 2012; Cheng et al., 2012; Wang et al., 2014; Tu et al., 2017; Lin et al., 2018, 2023; Chen et al., 2020). Therefore, fluids/sediments collected from mud pools are perhaps the best candidate materials that could be used to constrain the source characteristics and microbial communities inhabiting great depths, even though various degrees of modifications could have occurred during fluid ascending or in contact with the atmosphere. In this study, we combined the data reported in Tu et al. (2022) and demonstrated contrasting geochemical characteristics detected in different regions/sites. Fluids in the mud pools at sites AR and COM were reducing (Eh = −230 to −180 mV), alkaline (pH 7.8 to 8.6) and moderate saline (230 to 250 mM Cl^−^), and contained high dissolved methane (~1,000 μM) with isotopic compositions (δ^13^C-CH_4_ = ~ − 45‰) indicating mixed microbial and thermogenic sources. In contrast, fluids in the mud pool at sites PA01 and PA02 were more oxidized (−160 to −32 mV), acidic (pH 6.3), and saline (~1,100 mM Cl^−^) and contained much lower concentrations of dissolved methane (tens of μM) (Figure 2). These geochemical and field characteristics are consistent with the observations reported in previous studies and are closely associated with the control of geological settings (Cheng et al., 2012; Wang et al., 2014; Lin et al., 2018, 2023; Donato et al., 2021; Tu et al., 2022). In southwestern Sicily, mud volcanoes are located at the fault extension in a foreland basin formed by the northward subduction of the African plate underneath the Eurasian plate. Thick pelagic sediments accumulated during Miocene–Pleistocene were buried at great depths where temperatures were sufficient to transform organic matter into methane, other hydrocarbons, and smaller organic compounds, and to likely dehydrate clay minerals with the release of fluids. The fluids and gases mixed with unconsolidated fine-grained sediments ascend along the fault fracture to the surface or near surface environments. Low helium isotopic ratios (0.02 Ra) combined with isotopic compositions of methane suggest that exsolved gases originating from crustal depths entrain a portion of microbial methane produced at shallow intervals (Donato et al., 2021). For comparison, mud volcanoes located on the southern flank of Mt. Etna are underlie by the Triassic-Miocene sedimentary formations formed during the closure of Paleo-Tethys (Branca et al., 2003). Strontium isotopic compositions combined with ionic abundances indicate that saline fluids from the mud volcanoes represent deep, sedimentary brines expelled directly along the fault with limited contribution from either seawater or shallow aquifer (Liotta et al., 2017). Furthermore, high helium isotopic ratios (>6 Ra) and hydrocarbon isotopic compositions suggest that magmatic helium is likely mixed with hydrocarbons generated by thermal maturation (Allard et al., 1997; Caracausi et al., 2003). The geological settings enable the distinction of high methane and moderately saline fluids in mud volcanoes within a foreland from low methane and hypersaline fluids in mud volcanoes related to magmatism.

While the geochemical characteristics of fluids retrieved from mud pools constrain the source characteristics at depth, the continuous discharge of these fluids from the pool enables the overflow of fluids and sediments toward low topography. At any specific location, the top porewater and sediments are directly exposed to the atmosphere during the cessation of fluid immersion, thereby facilitating evaporative loss of porewater and penetration of atmospheric oxygen into sediments (Cheng et al., 2012; Wang et al., 2014). Chloride was used to trace the potential modification of fluid chemistry caused by surface evaporation, considering its inertness to most abiotic and biological reactions. For the collected sediment cores, the chloride concentrations of the pool fluids were comparable to those for porewater retrieved from the greatest sampled depth, suggesting that deeply-sourced fluids not only channel along the fracture network but also percolate through the pore space toward shallow intervals. Furthermore, the chloride concentrations of the pool fluids were always lower than those of shallow porewater by various degrees. The most drastic change in chloride among the investigated cores occurred at 7.75 cm for the core from site PA02 where chloride concentrations were enhanced by ~70% compared with chloride for the mud pool (1,185 mM versus 2014 mM). Despite the prevalent enhancement at shallow depths, chloride concentrations decreased with depth and approximated the concentrations of the pool fluids at three out of four sites. Such concentration variations could be best explained by the interplay between surface evaporation and the upward percolation of fluids originating from depth, a pattern comparable with those for Taiwan mud volcanoes (Cheng et al., 2012; Tu et al., 2017). Solute concentrations that vary in a manner different from that of chloride or other biologically inert solutes could be considered to result from microbial reactions.

### 4.2 Metabolic inference based on geochemical characteristics

The geochemical characteristics of pore fluids indicate the compartmentalization of different metabolic characteristics into discrete depth intervals. For site AR, a low sulfate zone was sandwiched between two high sulfate zones, suggesting two sulfate reduction zones at depth of around 10 and 30 cm (Figure 2A). In contrast, the methane concentrations and carbon isotopic compositions peaked at 12 cm and decreased at shallower and deeper intervals. Considering that methane solubility is limited and its diffusivity is greater than those of ions (Phan et al., 2016), its concentration in pore space could be highly affected by the presence of bubbles entrapped during sample collection. In this regard, methane isotopic compositions may reflect methane related metabolism more accurately than abundances. The isotopic variations with depth suggest that methane oxidation occurred at 10–30 cm intervals where δ^13^C-CH_4_ values were the greatest. A small shift of the δ^13^C-CH_4_ value (≤5‰) toward smaller values suggests various contributions of either methanogenesis (for 0–10 cm) or deeply sourced fluid (for ≥30 cm) whose isotopic compositions are best represented by the bubbling fluids. The enhanced DIC concentrations and δ^13^C values further suggest that methanogenesis at shallow depths could be acetoclastic or methylotrophic as both pathways convert organic precursors into methane depleted in ^13^C and CO_2_ enriched in ^13^C. The variation pattern of sulfate at site COM was similar to that at site AR, again indicating the presence of two sulfate reduction zones. While the variation in methane concentration was comparable to that in sulfate concentration, the δ^13^C-CH_4_ values decreased with depth over the investigated depth range. The top δ^13^C-CH_4_ value was even greater than that of the bubbling fluid by 10%. This isotopic pattern is best interpreted as the methanotrophic zone at shallow depths overlying the methane production zone at depth. While the DIC concentrations remained nearly constant (18 ± 2 mM), the δ^13^C-DIC values decreased with depth. The co-varied methane and DIC isotopic compositions suggest internal cycling of methane within a restricted niche, a pattern resembling marine AOM zones in seeps (Reeburgh, 2007; Yoshinaga et al., 2014). Finally, the concentrations of dissolved Fe and Mn were below the detection limit. As the dissolved forms of these metals are predominantly composed of divalent ions, their absence suggests either the limited activity of metal reduction or the rapid and efficient cycling that is involved in the metabolic network composed of various other elements (e.g., C and S). The compartmentalization of geochemical characteristics described above is similar to that of mud volcanoes (MVs) in Taiwan [e.g., Lei-Gong-Hou (LGH) and Shin-Yan-Ny-Hu (SYNH) MVs] (Wang et al., 2014; Lin et al., 2018). In SYNMV, while aerobic methanotrophy was active in the muddy pool, aerobic methanotrophy and sulfide/pyrite oxidation in top sediments were succeeded with sulfate reduction, sulfate-dependent methanotrophy, and methanogenesis as the depth increased (Cheng et al., 2012; Lin et al., 2018). Similarly, metabolic zonation in LGHMV was represented by the iron or manganese dependent methanotrophy and methylotrophic methanogenesis as the depth increased (Cheng et al., 2012; Wang et al., 2014; Tu et al., 2017). In summary, the presence of multiple sulfidization fronts suggests that sulfide has to be reoxidized, potentially by metal oxides (often in solid forms), and cycled to other forms efficiently so the observed sulfate can be kept at a low level. Such cryptic sulfur cycling can be also linked with methane metabolisms, and iron reduction and formation of iron sulfide/oxide minerals, thereby accounting for the reduced level of dissolved Fe and Mn. Candidate reactions network includes sulfide/sulfur oxidation coupled with metal reduction, methane oxidation coupled with sulfate and metal reduction, and organotrophic sulfate and metal reduction. Regardless of sites, the metabolic stratification suggests combinative effects of surface evaporation, downward oxygen perfusion, ascending fluid migration, fluid constituents, and availability mineralogy on regulation of redox and geochemical gradients. Microbial community members possessing specific metabolisms proliferate by harvesting catabolic energy generated by such geochemical transition and by adapting to the redox gradient imposed by geochemical and geological contexts.

In addition to the first order variations in methane, sulfate, and DIC, ethane and propane profiles for site AR are worth further notification. The concentrations of the two hydrocarbons were high (up to 3 μM for ethane and 1.2 μM for propane) at ≤25 cm. These patterns were distinct from that for methane in that the ethane and propane concentrations varied by more than one order of magnitude whereas the methane concentrations varied by less than a factor of three over the same depth range. Such concentration variations directly suggest the production and consumption of these high hydrocarbons at 5–15 cm and 15–20 cm, respectively. The synthesis and cracking of high hydrocarbons primarily occurs at great depths (>1,000 m) where the temperatures are sufficient (>90°C) to stepwise degrade organic matters into smaller aliphatic hydrocarbons (Kietäväinen and Purkamo, 2015). The temperatures of the sites investigated in this study were within the ambient temperature range (30°C). Therefore, it is unlikely that the formation and consumption of ethane and propane at this depth range are mediated through thermal maturation. Previous laboratory studies have demonstrated that members of Deltaproteobacteria and archaeal *ca.* Syntrophoarchaeum can mediate the consumption of propane and butane with the reduction of sulfate (Musat, 2015; Singh et al., 2017) or nitrate (Wu et al., 2023) under anoxic conditions. To date, limited microbial generation of ethane and propane has been experimentally validated.

Unlike the sites distributed within the accretionary wedge, methane and sulfate concentrations at sites near the Mt. Etna (PA01 and PA02) were less than 0.2 mM and below the detection, respectively. Considering that the methane concentrations remained at a nearly constant level along depth, and no isotopic data were available, methane metabolic activity was likely to be low. In addition, concentrations of dissolved iron for PA02 ranged between 0.15 and 0.30 mM at ≤29 cm depth and were below the detection for the mud pool and sediments at greater depths. The enhanced concentrations of soluble iron in the upper half depth range suggest that microbial processes are the main drivers converting iron oxides into soluble ferrous iron.

Except for the metabolic inferences, the geochemical characteristics may also impose a control on the cell abundance in sediments. qPCR results demonstrated higher bacterial and archaeal gene abundances at site AR than at site PA02 (Figure 4). By comparing the geochemical characteristics between these two sites, methane, sulfate and chloride appear to be the most prominent factors to distinguish each other. Other characteristics, such as dissolved iron and manganese, higher hydrocarbons, and sediment TOC and TN, are not that as discriminative as the three factors described above. As stated above, high methane abundances with fluctuating sulfate concentrations along depth at site AR facilitate to generate a strong redox gradient driven by ascending, reducing methane and descending, oxidizing oxygen. Such a redox transition can be translated into the stratified metabolic zonation rendered by discrete community compositions. In other words, the communities specializing specific ecological functions are compartmentalized over a small range of depth, leading to the proliferation and accumulation of high cell abundance. The high-abundance pattern has been also observed in other terrestrial mud volcanoes and seeps, wetlands, and tidal sediments (Lin et al., 2014; Thomas et al., 2014; Lin et al., 2018). In contrast, the same geochemical factors do not vary much along depth for PA02. The redox gradient is conceivably much less than that at AR, thereby producing no interpretable metabolic zonation that may account for the low biomass. Another possible factor for contrast cell abundances is the stress associated with salinity. The chloride concentrations were between 200–300 mM at AR and 1,200–2,100 mM at PA02. While the former one is less than seawater salinity, the latter one is considered to be in the hypersaline range. Community members imposed by such an environmental factor would have to cope with the osmotic stress by producing biocompatible solutes (e.g., betaine) (Ventosa and Nieto, 1995) or regulating ion transport across membrane (Oren, 2002), thereby limiting the proliferation of diverse taxa. Finally, a significant portion of cyanobacteria (~20%) were detected at shallower depths (down to 10 cm) at site AR (Figure 3). These phototrophs are capable of synthesizing organic carbon, constituting the carbon and energy bases that can fuel downstream metabolisms and heterotrophy. Therefore, such a system can be likely characterized by a vigorous energy and material exchange driven by both phototrophy and lithotrophy that harnesses the energy from geological sources (e.g., methane), thereby sustaining high cell abundance.

### 4.3 Community compositions and functions

The community compositions were drastically different for sites with different geochemical characteristics. At site AR, Deltaproteobacteria, Chloroflexi, and Bacteroidetes predominated over other bacterial groups and constituted more than 60% of the total bacterial reads (Figure 3). Desulfobacterales-related members comprising ~30% of the reads are known to be sulfate reducers that use various electron donors (e.g., H_2_, organic acids) (Dyksma et al., 2018). Woesearchaeota and ANME-2a-2b appeared to be the dominant components of archaeal communities (summed to be greater than 70% of the reads in most samples). The abundances of ANME-2 16S rRNA genes ranged between 7.9 × 10^6^ and 3.8 × 10^7^ copies g^−1^ sediments along depth (Figure 4A). ANME-2a-2b related members are anaerobic methanotrophs commonly observed in marine cold seep environments (Knittel et al., 2005; Yanagawa et al., 2011). Previous studies combining fluorescence *in situ* hybridization (FISH), nanoscale secondary ion mass spectrometry (nano-SIMS), biomarker and molecular analyses have demonstrated that this group of archaea forms syntrophic partnerships with several groups within Deltaproteobacteria to metabolize methane with the reduction of sulfate (Boetius et al., 2000; Orphan et al., 2001; Knittel et al., 2005; Wegener et al., 2015). Their presence has also been detected in terrestrial, ferruginous or sulfidogenic mud volcanoes in Taiwan (Tu et al., 2017; Lin et al., 2018), lake sediments, and estuarine sediments where the sulfate content is low (Weber et al., 2017). The sulfate concentrations at site AR varied inversely with the methane isotopic compositions, generating a pattern similar to that commonly observed in marine seeps and some terrestrial MVs. Considering that dissolved iron and manganese were all below the detection limit, high abundances of ANME-2 groups and geochemical characteristics suggest that AOM processes could be most likely dependent on sulfate reduction even though sulfate concentrations (<0.4 mM) and variation magnitudes were orders of magnitude less than those commonly observed in marine settings. It is worth noted that that AOM can occur at low sulfate concentration (10–500 μM) or with electron acceptors other than sulfate in laboratory incubations (Beal et al., 2011; Timmers et al., 2016; Mostovaya et al., 2021). Given the predominance of Deltaproteobacteria sulfate and iron reducers, and ANME-2 throughout sediments, AOM could be sustained by sulfate formed by the oxidation of sulfide through cryptic sulfur cycling or iron and manganese oxides. The caveat of cryptic sulfur cycling is that iron oxides are generally abundant in most sedimentary environments, providing nearly unlimited capacity for iron reduction. Therefore, even a portion of reduced iron or manganese is sequestered as sulfide minerals and not re-oxidized back to the soluble form, sediments can still either fuel iron reduction (with methane or organic oxidation) or react with sulfide produced from sulfate reduction to drive the wheel of sulfur transformation.

Less than 10% of the archaeal reads and 5% of the bacterial reads were affiliated with Methanosarcinales (excluding ANME-2a-2b)/Methanomicrobiales and Methylococcales, respectively. The former two lineages are capable of using various substrates (e.g., H_2_ and acetate) for methane production under anoxic conditions (Anderson et al., 2009; Mand and Metcalf, 2019). Their presence at shallow depths is consistent with geochemical constraints, suggesting the importance of the microbial role in contributing to methane generation. Since methanogens were distributed throughout the core and particularly enriched at the core bottom, methane fueling AOM may originate from a combination of *in situ* methanogenesis and a deeper source yet to be identified. In contrast, Methylococcales-related sequences are classified as type I methanotrophs capable of oxidizing methane under oxic conditions. The proportion of these aerobic methanotrophs was high at shallow depths and declined at greater depths (Figure 3). The proliferation of these methanotrophs is apparently confined to the depth range where oxygen penetration is likely to occur. Although the proportions of reads related to aerobic methanotrophs were low in sediments, the abundances of anaerobic and aerobic methanotrophs reached similar orders of magnitude in shallow sediments, assuming no PCR bias (Figures 3, 4). Therefore, even though aerobic and anaerobic methanotrophy are presumed to be compartmentalized into discrete zones, the current data are not adequate to infer the relative contribution of either methanotrophic pathway to the control of methane removal and emission.

Despite the methane and sulfate related metabolism, a large proportion of bacterial and archaeal reads were related to Desulfuromonadales (within Deltaproteobacteria) (17.8% for the VB01 primer set and 15.3% for the VB02 primer set) and Woesearchaeota (33.1% for the VA01 primer set and 71.2% for the VA02 primer set), respectively. Strains related to Desulfuromonadales are capable of reducing iron or manganese oxides (Greene et al., 2009; Greene, 2014). The proportions of Woesearchaeota-related reads did not exhibit any significant fluctuation with depth regardless of the primer set used. A high proportion of reads within Woesearchaeota (>90%) was classified as Pacearchaeota, within which no culture representative was obtained. These sequences have been detected in a wide range of environments such as marine and lake sediments, cold seeps, soil, and geothermal fluids (Castelle et al., 2015; Ortiz-Alvarez and Casamayor, 2016). Metagenomic data suggest that some Pacearchaeota possess genes capable of fermenting proteinaceous compounds (Castelle et al., 2015). In addition, a significant proportion of the recovered bacterial reads were related to Bacteroidetes and Chloroflexi whose closest culture representatives are capable of fermenting or respiring organic matter (Suominen et al., 2021; Zhang et al., 2023). Overall, the community compositions combined with geochemical characteristics suggest that while thermogenic methane originating from deep sources is abundant, methanogenesis using H_2_/CO_2_, acetate, and methyl-compounds also contribute to the overall methane inventory. Both anaerobic and aerobic methanotrophy account for the methane removal at shallow depths. At least a portion of AOM was linked to sulfate reduction, a metabolic scheme similar to that in marine setting. Community members responsible for organic degradation appear to be abundant and taxonomically diverse.

Forementioned compartmentalized community patterns at site AR were also observed in SYNH and LGH in Taiwan, however, the community compositions among these sites were different (Chang et al., 2012; Cheng et al., 2012; Wang et al., 2014; Tu et al., 2017; Lin et al., 2018). Methanogens are primarily related to genera *Methanosaeta* within Methanesarcinales and *Methanocalcalus* within Methanomicrobiales at SYNH, and genera *Methanosarcina* and *Methanococcoides* within Methanosarcinales at LGH. Methanotrophy is mediated by ANME-1 at SYNH and ANME-2 at LGH. These community constituents for methane related metabolisms in Taiwan are distinct from those at AR at genus level. Similar patterns for other potential metabolisms (e.g., sulfur and organic metabolisms) are also observed, suggesting that different combinations of community members could mediate resembling metabolic functions. The results were also comparable with an integrated control of both biogeography and local environmental context on community diversity of mud volcanoes distributed across the Eurasian continent (Tu et al., 2022).

The community compositions and functions at site PA02 were drastically different from those at site AR (Figure 3B). The majority of the archaeal communities were composed of Halobacteriales regardless of the primer sets used (>70% of the total reads). Its abundance was relatively low in the bubbling pool (10.3%), high at <23 cm (>90%), and decreased to 27.6% at 31 cm. Within Halobacteriales, most reads were classified as Halobacteriaceae, a family with culture representatives capable of growing on organic matter under high salt conditions (Oren, 2014). The abundance of Halobacteriaceae declined with depth and exhibited the opposite trend to that of Pacearchaeota (Woesearchaeota) (Figure 3B). Furthermore, the decrease of Halobacteriales or Halobacteriaceae with depth was compensated with the increase of Thermoplasmatales and Thaumarchaeota with depth. The distribution patterns combined with relatedness with the culture representatives suggest that the detected community members might possess different affinities for oxygen. While the Halobacteriaceae-related members respire organic matters with oxygen, the Pacearchaeota, Thermoplasmatales, and Thaumarchaeaota-related members would be most likely anaerobes (Pester et al., 2011; Castelle et al., 2015). Like that described previously, metagenomics data suggest that some of the Pacearchaeota and Thermoplasmatales-related members are equipped with genes capable of fermenting proteinaceous compounds (Castelle et al., 2015). Therefore, their enhanced proportions suggest their possible role in fermenting organic matter under anoxic conditions. Several culture representatives and metagenomes have been obtained for Thaumarchaeota (Spang et al., 2010; Santoro et al., 2015). These taxa are known of autotrophically oxidizing ammonium under oxic conditions and distributed prevalently in global soils and seawater (Tourna et al., 2011; Löscher et al., 2012; Santoro et al., 2015). Whether their decline abundance with depth at PA02 suggests an electron acceptor other than dioxygen warrants further investigation. A fraction of Methanomicrobiales and Methanosarcinale-related members (<5%) and anaerobic methanotrophs (ANME-2a-2b) (0.2%) were detected, suggesting limited potential for methanogenesis and AOM. Compared to the core samples, the Halobacteriales were much less abundant in the mud pool. Instead, Methanosarcinales (27.1%) and Thermoplasmatales (19.5%) became dominant taxonomic orders. Because pool fluids originate from a deep source, such a community composition would represent community compositions at great depths. By comparing the archaeal communities in the bubbling pool with those in the cored sediments, a transition from the predominance of methanogens to Halobacteriales suggests a strong impact of atmospheric oxygen on modifying community composition and function. Methanogenesis could be active in deep fluid reservoirs, become inhibited upon exposure to the atmosphere, and be possibly re-activated until the sediments are buried to a depth where oxygen is scarce and methanogenic precursors are available. The exposure of deeply sourced fluids and sediments to the atmosphere, however, facilitates the proliferation of oxic, halophilic heterotrophs related to Halobacteriaceae.

The bacterial community composition at site PA02 was also distinct from that at site AR. Gammaproteobacteria, Deferribacteres, Deltaproteobacteria and Chloroflexi were the major phyla/classes. In particular, the proportion of reads related to Gammaproteobacteria was approximately 40% regardless of the primer sets used. Among the various taxa within Gammaproteobacteria, orders Methylococcales and Thiotrichales, and genera *Thiohalophilus* and *Thiohalorhabdus* appear to be the most abundant taxonomic units. In core samples, *Thiohalorhabdus*-related members dominated over the others. The culture representatives affiliated with this lineage are known to oxidize sulfur species (e.g., sulfide, thiosulfate, and tetrathionate) via the reduction of oxygen or nitrate in high salinity environments (Sorokin et al., 2008). The physiological characteristics of Thiotrichales and *Thiohalophilus*-related members generally resemble those of *Thiohalorhabdus* (Sorokin et al., 2007). For comparison, Methylococcales-related members are known to oxidize methane under oxic conditions (Wrede et al., 2012). While the abundances of these four lineages generally decreased with depth, their activities could have been largely controlled by oxygen. The abundance pattern of Deferribacteres was similar to that of Gammaproteobacteria, whereas that of Chloroflexi and Deltaproteobacteria fluctuated over the investigated depth range. The reads classified into the Deferribacteres and Chloroflexi phyla were all related to environmental sequences retrieved from various environments. They are generally considered to be the fermenters responsible for the breakdown of complex molecules into smaller organic acids (Rinke et al., 2013; Tu et al., 2017; Suominen et al., 2021). Within Deltaprotoebacteria, Desulfarculales, Desulfobacterales and Desulfuromonadales were the most abundant orders. While the former two orders are composed of strains capable of reducing sulfate (Kuever et al., 2005; Wasmund et al., 2017), strains affiliated with the last order are primarily metal reducers (Mehta et al., 2005). Since the concentrations of sulfate and dissolved iron and manganese were all below the detection limit (Figure 2D), their low abundances might reflect limited metabolic activities. Finally, a notable feature is that a considerable proportion of bacterial reads (~1.5%) were classified into one OTU affiliated with *Mariprofundus* within Zetaproteobacteria. The abundance of this OTU increased from about 0.5% at the sediment top to between 1.3 and 2.0% at 4 cm, and declined steadily to less than 0.3% at the bottom of the cored sediments. Strains affiliated with this genus can oxidize ferrous iron at neutral pH (Emerson et al., 2007). Their peak abundances at 4 cm suggest their preference for an oxygen level over than that in equilibrium with atmospheric oxygen. Considering that the soluble iron concentrations were below the detection limit, the exact metabolic activity mediated by the strains represented by this OTU remains to be addressed.

The community patterns for sites AR and PA02 were distinct from each other regardless of the primer sets used. However, different primer sets induced different degrees of bias toward specific groups of communities (e.g., Methanosarcinales). While the specificity has been tested *in silico* (>94% for archaea), the low coverage might arise from the experimental conditions that could not ideally render the primer performance as good as the *in silico* examination suggests. The primer combinations used in this study were initially tested on several samples by adjusting the annealing temperature across a gradient. The temperature with the highest yield was subsequently chosen for further PCR on all the other samples. Therefore, it is likely that such a selection on experimental conditions did not enable the high recovery of Methanosarcinales. In addition, the VA02 primer set targets highly variable regions, V4-V6. Even the specificity derived from *in silico* examination against the existing sequences in the database is high, the Methanosarcinales members may possess a sequence with priming sites different from the VA02 primer set. Although this primer set has been used for various environments, the limited coverage of taxa amplified by the VA02 primer set would be particularly disadvantageous for studies on methane-rich environments where methanogens using acetate or methanol as substrates, and ANME-2 and -3 groups are more abundant. The amplification bias could lead to reduced sensitivity in recovering target microorganisms involved in methane related metabolism. In contrast, the two bacterial primer sets yielded similar results, even though the abundances of specific lineages varied (Figure 3).

The PCoA analyses showed that both archaeal and bacterial communities at sites AR and PA02 were distinct, and the variance was significantly associated with chloride concentration. The analyses with VA01 primer set further revealed that methane significantly contributed to the archaeal community variance at site AR (Figure 5A). Furthermore, the archaeal and bacterial communities at site AR clustered regardless of the depth intervals recovered, suggesting that the vertical gradient in geochemical characteristics did not exert a profound impact on community compositions (Figure 5). In contrast, archaeal and bacterial communities at site PA02 varied considerably along depth. The archaeal communities in the pool fluids (ew) and at 22.8 and 31 cm depth were separated from a cluster formed by communities at shallow depths along PC2. Such variations were primarily caused by the reduced predominance of Halobacteriales and increasing proportions of Thermoplasmatales, Thaumarchaeota and unidentified Euryarchaeota (Figure 3B). The resemblance between the pool and deepest core archaeal communities further suggests that the deeply-sourced archaeal members are resistant to the atmosphere exposure so their functions would be re-activated once the sediments are buried to a certain depth interval where the geochemical context becomes favorable for these archaeal members. The variations in bacterial communities at site PA02 were also large along PC2 but proceeded in a trend not exactly the same as those in archaeal communities. First, the bacterial community in the mud pool was greatly separated from the communities in the core primarily because of the enhanced proportions of Campylobacterota and Triotrichales within Gammaproteobacteria (Figure 3B). Second, the bacterial community variations in the core trended with depth, a pattern primarily controlled by the decreasing Gammaproteobacteria and Deferribacteres with depth. The communities at two deepest intervals deviated at the greatest degree from that in the mud pool, suggesting the profound modification of community compositions during the exposure to the atmosphere and burial processes in the mud platform.

## 5 Conclusion

Geochemical and molecular analyses of four mud volcanoes and seeps in Sicily, Italy suggest that their geochemical characteristics, community compositions and functions are controlled by the geological context. At the sites within the accretionary wedge where methane and sulfate were abundant, community members related to methane and sulfate metabolism (such as ANME-2, Methylococcales, and Desulfobacteriales) and a number of taxa without any culture representative (such as Pacearchaeota) or involved in organic degradation (such as Chloroflexi and Desulfuromonadales) constituted a significant proportion of communities. The prevalence of these taxa and the relatively low community variation with depth suggest the importance of anaerobic and aerobic methanotrophy in controlling methane release from terrestrial mud volcanoes where material cycling and fluid transport are largely related to subduction processes. The results also indicate that sulfate reduction was partly involved in methane oxidation, a metabolic scheme commonly found in marine cold seeps. In contrast, the sites near Mt. Etna were characterized by high salinity and CO_2_, and low methane and sulfate. The community compositions were composed of halophilic organic degraders (such as Halobacteriaceae, Deferriberes, and Chloroflexi) and sulfur metabolizers (such as *Thiohalophilus* and *Thiohalodbdus*). A minor fraction of aerobic methanotrophs (e.g., Methylococcales) were also detected. The depth variation in community composition suggests that the local physicochemical context imposes a strong control on the community compositions and functions. Instead of anaerobic methanotrophy, methanogenesis, and sulfate reduction, community members capable of oxidizing organic compounds and sulfur dominated over the others. Overall, the geological context combined with the fluid path and source characteristics drives the proliferation of specific community members at different depth ranges and sites. The distinct community patterns and compositions also highlight that a wide spectrum of community compositions and functions are involved in carbon and sulfur cycles.

## Data Availability

The datasets presented in this study can be found in online repositories. The names of the repository/repositories and accession number(s) can be found in the article/Supplementary material.

## Footnotes

^1^http://www.arb-silva.de
