# Supplementary figures and images for: Variations in microbial community compositions and processes imposed under contrast geochemical contexts in Sicilian mud volcanoes, Italy
Source: Front Microbiol. 2024 Sep 20;15:1461252. doi: 10.3389/fmicb.2024.1461252 (PMC11449744; doi:10.3389/fmicb.2024.1461252)

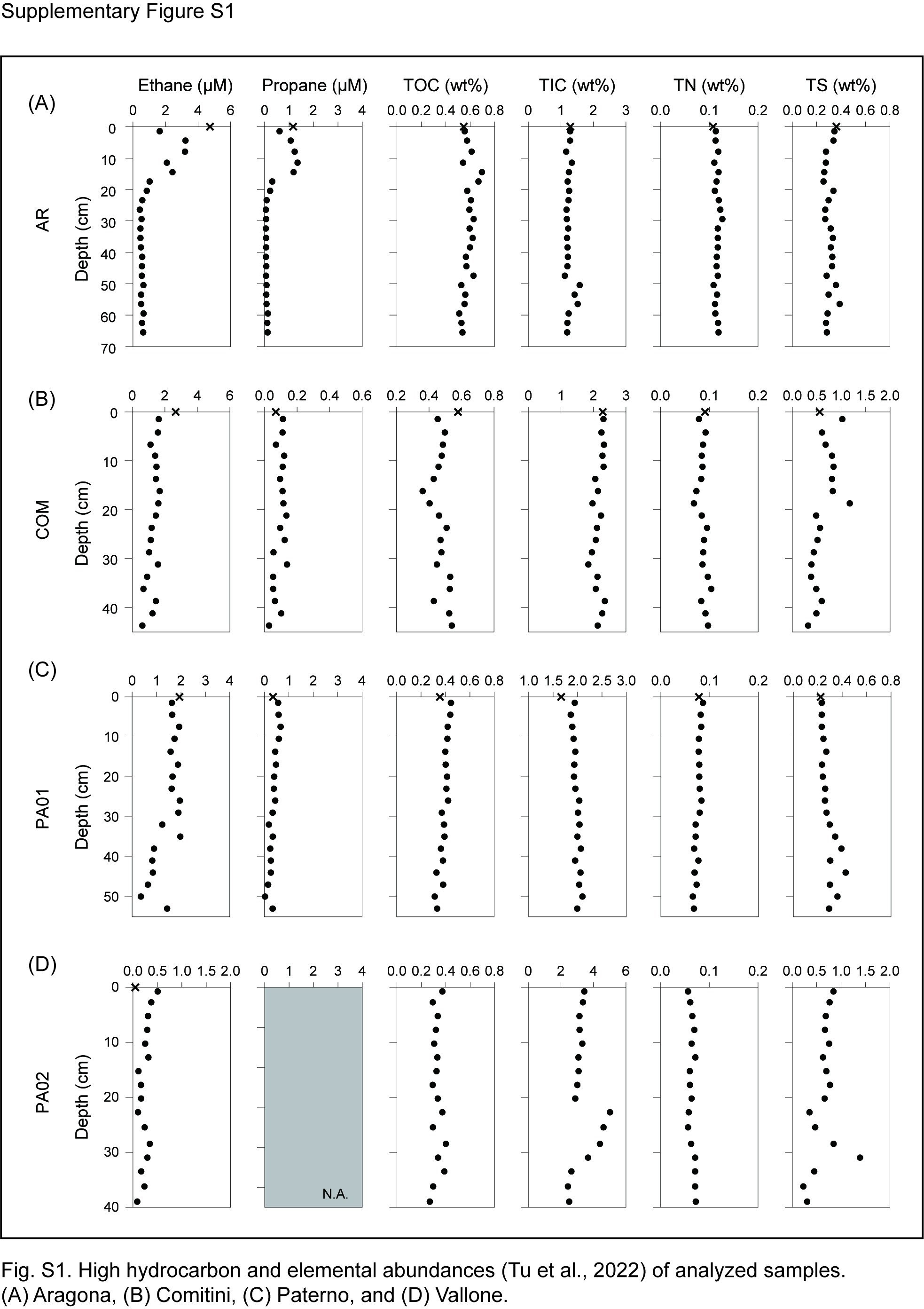

Supplement: Supplementary file 1 [file Image_1.TIF]

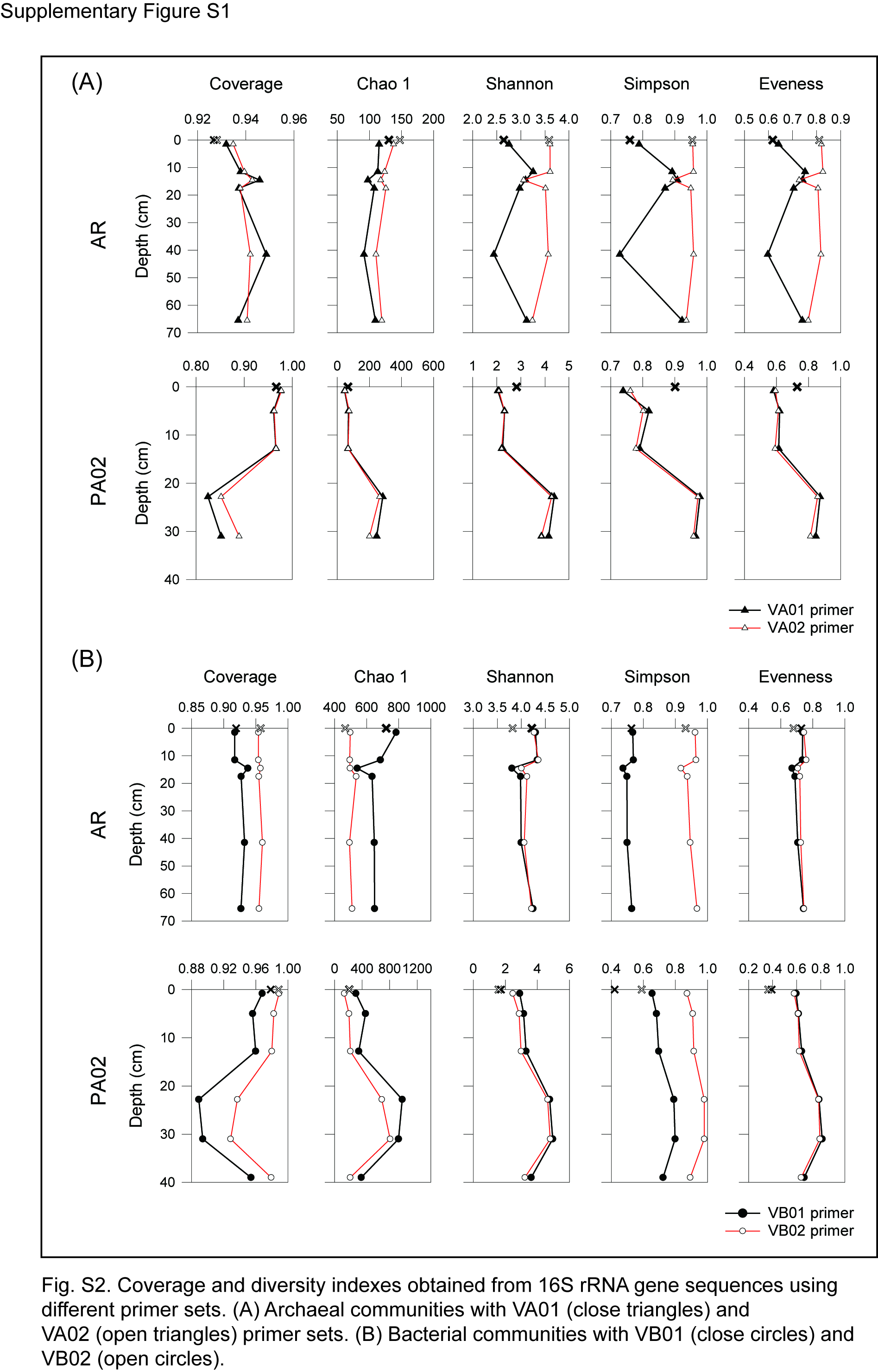

Supplement: Supplementary file 2 [file Image_2.TIF]
