# Supplementary material for: Variations in microbial community compositions and processes imposed under contrast geochemical contexts in Sicilian mud volcanoes, Italy
Source: Front Microbiol. 2024 Sep 20;15:1461252. doi: 10.3389/fmicb.2024.1461252 (PMC11449744; doi:10.3389/fmicb.2024.1461252)
Supplement: Supplementary file 3 [file Table_1.DOCX]

Table S1. Read and OTU counts obtained from individual primer sets.

| Target group | Archaea | | | Bacteria | | |
| --- | --- | --- | --- | --- | --- | --- |
| Primer set | VA01 | VA02 | Sum | VB01 | VB01 | Sum |
| Aragona (AR) | | | |  | | |
| Total reads | 16,334 | 30,339 | 46,673 | 20,826 | 22,989 | 43,815 |
| OTU numbers | 321 | 509 |  | 1,185 | 785 |  |
|  | | | |  | | |
| Vallone (PA02) | | | |  | | |
| Total reads | 21,786 | 25,360 | 47,146 | 21,657 | 27,661 | 49,318 |
| OTU numbers | 648 | 543 |  | 1,329 | 893 |  |

*The detailed information for individual primers and primer sets are described in the main text.
