# Supplementary material for: Variations in microbial community compositions and processes imposed under contrast geochemical contexts in Sicilian mud volcanoes, Italy
Source: Front Microbiol. 2024 Sep 20;15:1461252. doi: 10.3389/fmicb.2024.1461252 (PMC11449744; doi:10.3389/fmicb.2024.1461252)
Supplement: Supplementary file 5 [file Table_3.DOCX]

Supplementary information

The cores and DNA extracts we used in this study are the same as those used in Tu et al. (2022). Two representative cores collected from sites AR and PA02 were used for gene analyses. This selection was based on the geochemical data reported by Tu et al., (2022) and this study, in which the sites AR and COM could be categorized from the other cluster encompassing sites PA01 and PA02. As indicated by Tu et al. (2022), community variance based on amplicon sequences generated by the universal primers is dependent on the local geochemical context and geographic distance between the samples and cores across the Eurasian continent. At a local scale, sites AR and COM can be geochemically distinguished from the other cluster composed of sites PA01 and PA02 by methane, sulfate, and chloride concentrations (Fig. 2). The beta diversity between sites AR and COM or between sites PA01 and PA02 is lower than that between sites classified by distinct geochemical categories or larger geographic distances. The alpha diversity patterns can also be categorized in terms of geochemical characteristics (Fig. S4 in Tu et al. 2022). Finally, the variance analyses reported by Tu et al. (2022) show that the communities of AR and COM resemble each other to a higher degree than those of PA01 and PA02. These lines of evidence suggest that replicate core communities from a similar geochemical context at a local scale might not be needed to address the community variance. Therefore, one representative core from individual sites AR and PA02 was selected for detailed analyses of community compositions and functions.
